# Fast genome-based delimitation of Enterobacterales species

**DOI:** 10.1371/journal.pone.0291492

**Published:** 2023-09-14

**Authors:** Julie E. Hernández-Salmerón, Tanya Irani, Gabriel Moreno-Hagelsieb

**Affiliations:** Department of Biology, Wilfrid Laurier University, Waterloo, ON, Canada; University of Bologna / Romagna Local Health Authority, ITALY

## Abstract

Average Nucleotide Identity (ANI) is becoming a standard measure for bacterial species delimitation. However, its calculation can take orders of magnitude longer than similarity estimates based on sampling of short nucleotides, compiled into so-called sketches. These estimates are widely used. However, their variable correlation with ANI has suggested that they might not be as accurate. For a where-the-rubber-meets-the-road assessment, we compared two sketching programs, mash and dashing, against ANI, in delimiting species among Esterobacterales genomes. Receiver Operating Characteristic (ROC) analysis found Area Under the Curve (AUC) values of 0.99, almost perfect species discrimination for all three measures. Subsampling to avoid over-represented species reduced these AUC values to 0.92, still highly accurate. Focused tests with ten genera, each represented by more than three species, also showed almost identical results for all methods. *Shigella* showed the lowest AUC values (0.68), followed by *Citrobacter* (0.80). All other genera, *Dickeya*, *Enterobacter*, *Escherichia*, *Klebsiella*, *Pectobacterium*, *Proteus*, *Providencia* and *Yersinia*, produced AUC values above 0.90. The species delimitation thresholds varied, with species distance ranges in a few genera overlapping the genus ranges of other genera. Mash was able to separate the *E. coli* + *Shigella* complex into 25 apparent phylogroups, four of them corresponding, roughly, to the four *Shigella* species represented in the data. Our results suggest that fast estimates of genome similarity are as good as ANI for species delimitation. Therefore, these estimates might suffice for covering the role of genomic similarity in bacterial taxonomy, and should increase confidence in their use for efficient bacterial identification and clustering, from epidemiological to genome-based detection of potential contaminants in farming and industry settings.

## Introduction

Average Nucleotide Identity (ANI), is becoming a standard in genome sequence-based species delimitation [1–5]. The method involves cutting a genome sequence into segments, commonly around 1000 base-pairs long, to compare them against the full sequence of another genome. As the name implies, the calculation consists on the average identity of matching segments. While much faster than experimentally-based approaches, such as DNA-DNA hybridization, the method can take a very long time to compare thousands of genomes, even when using an optimized program for the calculation, such as fastANI [6].

Methods based on sampling k-mers, normally between 20 and 40 bp long, can produce genome similarity/distances orders of magnitude faster than ANI [7–9]. The most commonly used of these methods is the MinHash approach, implemented into the mash software [7]. This program gathers k-mer samples, codified as hashes, into so-called sketches, which can be efficiently compared. The overall genome similarity is then estimated from the proportion of identical k-mers found between the compared sketches. MinHash can estimate genome similarities in a fraction of the time required for ANI [7, 8], and have been reported to correlate well with ANI measurements [7]. Another program, dashing [9], offers a few different approaches to codifying sketches, and can produce results very similar to those by mash, among other useful genome similarity estimates, such as a Jaccard index [9].

While the articles on mash and dashing show that their results correlate with those produced by ANI [7, 9], the correlations can vary in measure [6]. Therefore, more where-the-rubber-meets-the-road analyses seem to be required to properly evaluate how well they would substitute calculations of overall genome similarity. We recently published a comparison of these programs against ANI in delimiting species in the *Klebsiella* genus [8]. Since the classification of *Klebsiella* has involved ANI along other features [10–12], it is not surprising that ANI-based clusters had an excellent match with species annotations in this genus. The good news was that the sketch-based methods produced results of the exact same quality [8].

Here we present an expansion of those analyses towards all of the complete genomes available at NCBI’s RefSeq genome database [4], classified into the Enterobacterales taxonomic order, to try and ensure that the sketch-based estimates can work as well as ANI in delimiting species beyond those of *Klebsiella*. We therefore tested fastANI, mash and dashing for delimiting species at the overall order level, as well as within genera represented by more than three species.

## Methods

### Genome sequences

To compile a genome sequences dataset, we downloaded all available genomes with a “Complete” status at NCBI’s RefSeq genome database [4], by the middle of January of 2023. From these, we selected all the genomes classified into the order Enterobacterales, as long as they were classified to the species level, for a total of 7,132 genome sequences (S1 Table). Type strains were identified from the annotations found in the corresponding NCBI’s “GBFF” files. The assembly identifiers of these genomes is included in the Supporting information tables. These identifiers can be used to download the corresponding sequences from NCBI.

### Genome comparisons

To calculate Average Nucleotide Identity (ANI), we selected the fastANI program [6], produced by the group that, as far as we know, has most promoted the use of ANI in genome-wide classification studies [6, 13–15]. Briefly, the original ANI calculations [16], started by breaking a query genome into 1020 base-pair long segments, which were then compared against a target genome using NCBI’s blastn [17]. As the name suggests, ANI was then calculated by taking the average identity of segments matched by blastn. Instead of blastn, fastANI uses a MinHash approach to quickly estimate the similarity between the genome-derived DNA segments and the target genome [6]. Running comparisons with fastANI’s default fragment length of 3000 bp left many genome pairs without calculated similarities. Thus, we used a fragment length of 1020 (--fragLen 1020), which corresponds to the fragment length used in most of the original implementations of ANI. Every other option was left at its default value. The ANI produced by fastANI is reported as a percent identity. Therefore, to transform ANI into distances, we subtracted the obtained ANI from 100 and divided the result by 100.

To calculate distances using the MinHash approach, we used two programs: mash [7] and dashing [9]. For mash, version 2.3, we produced sketches of 5000 k-mers (-s 5000 option), rather than the default of 1000. Since these distance estimates depend on the number of k-mers found in common, a higher sketch size should result in better representation, thus better estimates. Accordingly, our preliminary tests showed that the default failed to produce distances for many genome pairs. We did not use sketch sizes of more than 5000, because our preliminary tests also found that the results were almost identical to those produced using sketches sizes of 10,000. Every other option was left to its default value. The distances produce by mash are reported as fractions, rather than percents.

We used dashing version 1.0.2. We changed the k-mer length to 21 bp, instead of its default of 31, for two reasons: (1) because 21 is the k-mer size used by mash, and (2) with 31 bp, the program failed to estimate distances for several genome comparisons. We used a sketch size of 2^14^ (-S 14), because this sketch size produced the best estimates of genomic Jaccard similarity in the original dashing publication [9]. We only tested mash distances (-M option) because our prior report showed that results from other measures were identical [8]. Every other option was left unchanged from its default value.

### Accuracy and plotting

Plots and other calculations were produced using R [18]. To test the accuracy of all programs, we performed Receiver Operating Characteristic (ROC) curve analyses as implemented in the R package cutpointr [19]. We also used the cutpointr package to find optimal cutoffs. Hierarchical clusters were displayed using the R packages ggtree [20] and ggtreeExtra [21].

## Results and discussion

Though the focus of this report is on the quality of results, it is noteworthy that calculating ANI between all genomes, with the fastANI program, required the separation of all genomes into several datasets to divide the labour across three computers. The calculation took around two months. Calculating distances using mash or dashing took no more than a couple of hours, including the time to produce “presketch” files, which can be reused to produce results in a more timely manner. These fast programs were run in an iMac computer with 32G of RAM and an Intel processor. The programs ran using four of the available 8 cpu threads.

### All methods discriminated Enterobacterales species with high accuracy

**Fig 1:**
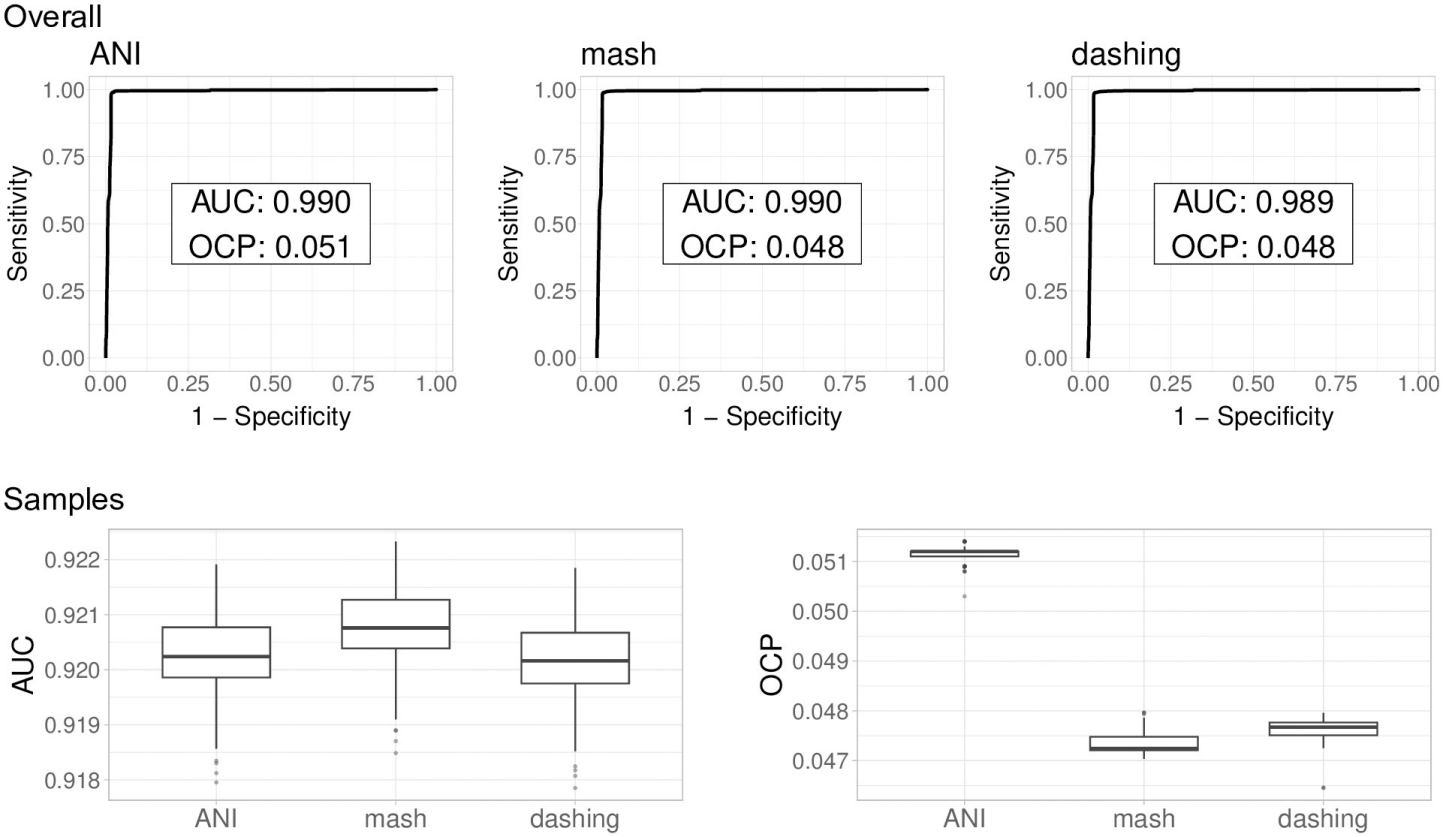
Species separation across Enterobacterales. All three methods produced exactly the same overall quality of species separation with Area Under the Curve (AUC) values close to 0.99. The optimal cutoff points (OCP) for species separation suggested by the analysis are slightly lower for the sketching algorithms (mash and dashing). Differences between estimated distances and full genome based distance calculations are to be expected. Because mash and dashing use the same formula, though different methods for k-mer representation, it is expected for them to produce similar estimates. After subsampling genomes from over-represented species, to avoid bias, the AUC lowered to a median slightly above 0.92 with all programs, with OCP similar to those obtained with the full set of genomes.

To test species/genus separation using all three tools, fastANI, mash and dashing, we produced tables with all *vs*. all pairwise distances, as calculated with all three methods (S2 Table). Pairs belonging to either the same species, or the same genus / different species, were used to test for species discrimination. A test with all same species and same genus pairs showed AUC values above 0.99 for all three methods (Fig 1).

The species representation of the Enterobacterales genome dataset is heavily biased. The dataset contains a number of *Escherichia coli* representatives orders of magnitude larger than those of most other species. Therefore, the AUC values might be biased towards the distinction of *E. coli* (of all 4,381,875 same species pairs, 2,671,516, or 61%, are *E. coli* pairs). The abundance of *E. coli*, *Klebsiella pneumoniae* (944,625 pairs, 22%) and *Salmonella enterica* (697,971 pairs, 16%) covered 99% of all same species pairs, even though the dataset contained 256 species, with 139 of them represented by more than one genome.

To avoid the effects of these biases, we obtained 100 subsamples each containing 100 genomes randomly sampled from all species represented by more than 100 genomes. This leveled the number of the top five most represented species. All other species kept their total genomes, with several of them having representative numbers within the same order of magnitude of the subsamples. Again, all three methods produced results of the same quality, though the median AUC values were slightly above 0.92 (Fig 1), lower than the AUC values obtained in the naïve test above, but still indicating very good accuracy in species separation.

Prior results suggested that MinHash, represented by mash, was not as accurate as ANI [6]. However, “accuracy” in that report was measured in terms of Pearson correlation against ANI, as traditionally calculated, rather than about actual accuracy in the task of delimiting species [6].

### Different genera showed different within-species distances with all methods

The tests above combine all same species and same genus pairs. However, the most difficult distinction should be between genomes from organisms of the same species from within the same genus. Therefore, we ran further tests choosing genera from the Enterobacterales, represented by at least three species, making sure that all three species within the selected genera were represented by ten genomes or more. This process resulted in ten genera: *Citrobacter*, *Dickeya*, *Enterobacter*, *Escherichia*, *Klebsiella*, *Pectobacterium*, *Proteus*, *Providencia*, *Shigella*, and *Yersinia*.

Of the ten genera selected above, two had severe biases in abundance of genomes representing a single species: *Escherichia* and *Klebsiella*. As above, to avoid biases due to over-represented species, we sub-sampled genomes from the most represented species to level them with the second most abundantly represented species.

*Klebsiella* is the genus with the best species representation of all ten, with seven species represented by more than ten genomes, six of them by more than 30. *K. pneumoniae* exceeded the representation of the next more represented species, *K. quasipneumoniae* by an order of magnitude. We therefore produced 50 subsamples topping 50 representative genomes per species, thus leveling the order of magnitude in representation for the top six genomes. The AUC for species discrimination in this genus remained close to 0.99 in all subsamples with all programs tested (Table 1). As explained above, ANI has been part of the taxonomic assignments in the genus [10–12], which explains the high accuracy obtained with this measure. It is still important, however, to note that mash and dashing produced results of the same quality. We have presented a more focused work about species delimitation in this genus before [8].

**Table 1:** Area under the curve (AUC) values for species/genus separation across ten genera.

Genus	No. species	ANI	mash	dashing
*Citrobacter*	14	0.805	0.802	0.804
*Dickeya*	9	0.999	0.999	0.999
*Enterobacter*	14	0.921	0.921	0.922
*Escherichia* *	4	1.000	1.000	1.000
*Klebsiella* *	11	0.987	0.988	0.987
*Pectobacterium*	16	0.964	0.965	0.963
*Proteus*	6	0.995	0.995	0.995
*Providencia*	7	0.971	0.972	0.954
*Shigella*	4	0.690	0.673	0.677
*Yersinia*	17	0.990	0.990	0.989

* The values for *Escherichia* and *Klebsiella* are median AUCs calculated from 50 subsamples.

As expected from *E. coli*’s overall over-representation among Enterobacterales, *E. coli* was represented by orders of magnitude more genomes than the next *Escherichia* species, *E. fergusonii*. We therefore produced 50 subsamples of the genus, topping the number of representative genomes per species to 50, thus leveling the order of magnitude of three of the four species in this genus. The species separation in all these subsamples was always clean (AUC: 1).

**Fig 2:**
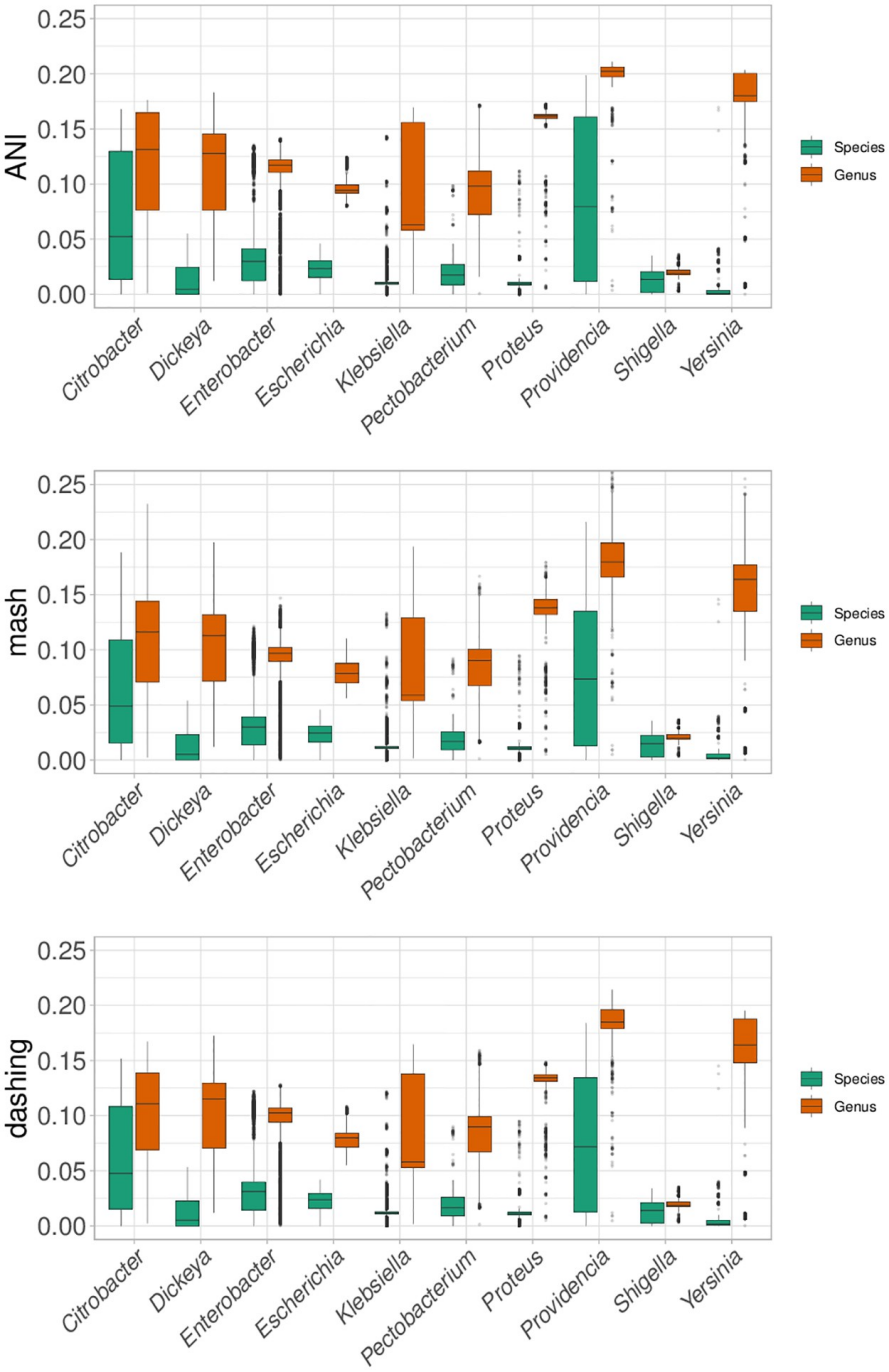
Species distances. The range of species distances varied from one genus to the next, suggesting that there is no universal species delimitation. Though results are very similar for all measures, dashing seemed to produce more outliers.

Distance ranges varied among the species in the ten genera (Fig 2). *Yersinia* showed the lowest values, while *Providencia* showed species values overlapping the within-Genus distributions of most other genera. This variation agrees with a previous study focused on the ANI distributions of several bacterial species [22], but contradicting the notion of a universal species delimitation threshold [6, 23]. This result also seems to conflict with the high accuracy for species delimitation across all Enterobacterales that we presented above. However, the apparent conflict is resolved by the fact that the species in seven of these genera have distance ranges below, roughly, 0.05, combined with within-genus distance ranges above the same mark.

In summary, the AUC values for species discrimination among all ten genera varied (Table 1). The lowest values obtained were for *Shigella*, with AUC values of approx. 0.68. That value was followed by 0.80 for *Citrobacter*. Most other AUC values were above 0.90 (Table 1). Again, the results were almost identical with all three measures.

### The *E. coli* + *Shigella* complex separated into 25 phylogroups

**Fig 3:**
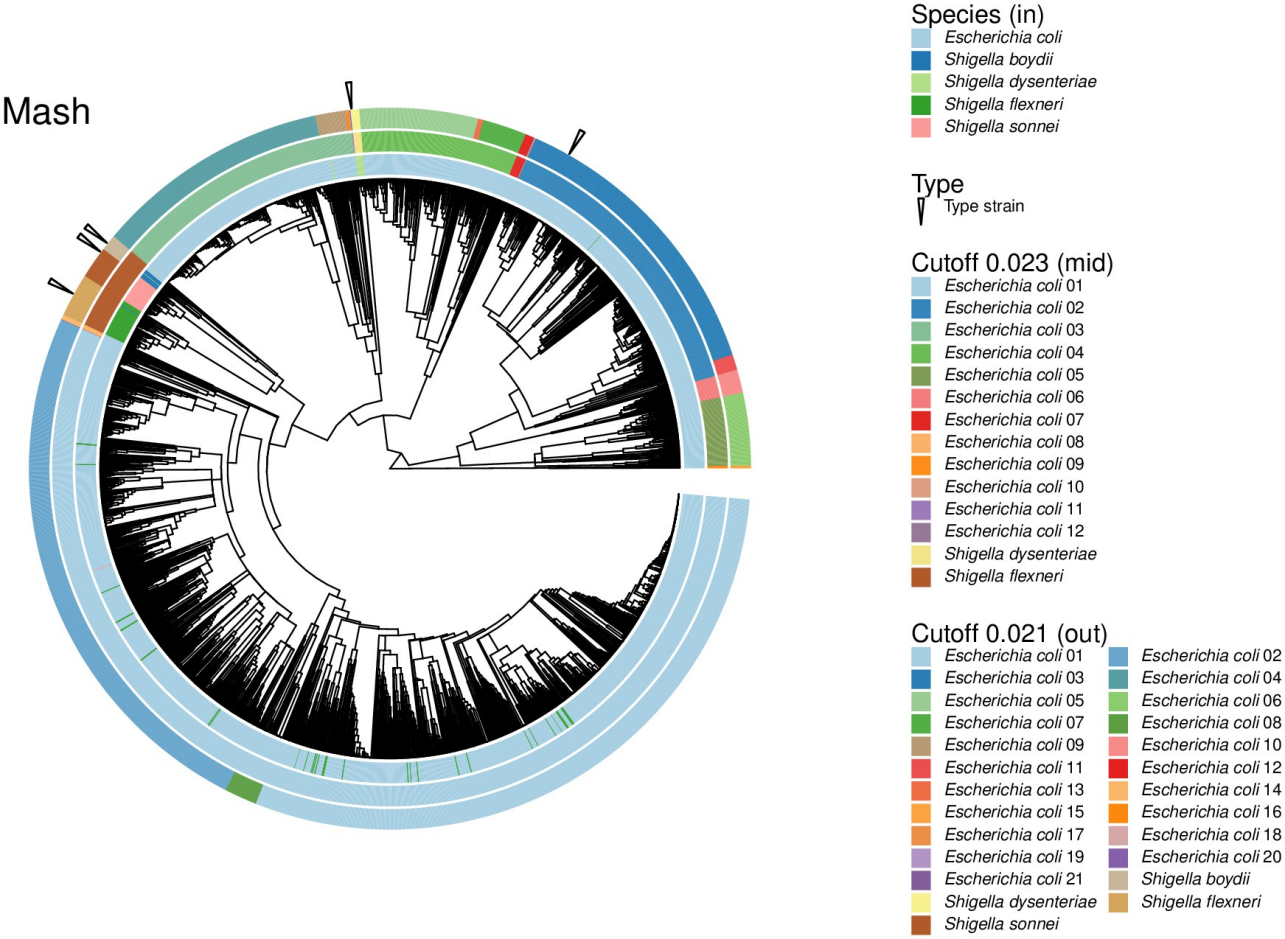
The *Escherichia coli* + *Shigella* complex. The hierarchy shows that several *Shigella* genomes clustered together forming coherent groups, though many are interspersed into groups dominated by genomes labeled as *E. coli* (inner circle, or circle closest to the dendogram). A mash distance cutoff of 0.023 produced 14 groups, two of them mainly composed of *Shigella* genomes (middle circle). A lower cutoff, 0.021, separated *Shigella* into all four represented species, but it also divided the whole group into a total of 25 clusters (external circle), or apparent phylogroups.

As explained above, while the separation of species in the *Escherichia* genus was clean for all subsamples tested (AUC: 1), that for *Shigella* was the worst (AUC: 0.68). However, the clustering of *Shigella* within the *E. coli* species is a classic taxonomy *vs*. tradition issue (discussed in [24], and reanalysed in [25]). Accordingly, cutting hierarchical clusters at an ANI distance of 0.051, or a mash distance of 0.048 (the cutoffs suggested for the whole dataset when optimising for the F1 score using the cutpointr package; Fig 1), produced a cluster containing all *E. coli* genomes combined with all the genomes representing the four *Shigella* species in the dataset (Fig 3). No other species in Enterobacterales, belonging to different genera, showed such within-species distances in our dataset. We examined this group more closely.

Examination of the hierarchical cluster produced from either distance measure showed some clean groups composed of *Shigella* species (Fig 3, inner circle). However, other *Shigella* strains were interspersed with clear *E. coli* groups. Similarly, a few genomes annotated as *E. coli* were inserted into *Shigella* groups. These two cases suggest the presence of some mislabeled genomes (Table 2). Therefore, it is possible that the anomalously clustered genomes need to be relabeled according to the main names within their apparent phylogroups. To test for this possibility, we looked for a mash distance cutoff that could cleanly separate the observed clusters that coincide with *Shigella* species. A mash distance cutoff of 0.021 worked for this purpose. In other words, this cutoff produced four *Shigella* clusters, each corresponding to a *Shigella* species, and each containing their corresponding type strain (Fig 3, outermost circle; S3 Table). Two of these were clean single-species groups: *S. sonnei* and *S. dysenteriae*. The *S. flexneri* group contained an apparently mislabeled *S. boydii* genome, while the *S. boydii* group contained five *E. coli* genomes and two *S. dysenteriae* ones. This cutoff produced 21 *E. coli* groups, for a total of 25 apparent phylogroups (Fig 3, outermost circle; S3 Table). Of these, 17 were clean *E. coli* clusters. Three of the four apparently contaminated *E. coli* groups were mixed with *S. flexneri*, with one of them also containing a genome labeled as *S. sonnei*. The remaining *E. coli* group contained two genomes labeled as *S. dysenteriae*.

**Table 2:** Potentially mislabeled genomes among the apparent 25 phylogroups in the *E. coli* + *Shigella* complex.

Assembly ID	Species label	Group
GCF_002949935	*Shigella dysenteriae*	*Shigella boydii*
GCF_002950015	*Shigella dysenteriae*	*Shigella boydii*
GCF_007197595	*Shigella flexneri*	*Escherichia coli* 03
GCF_012221365	*Escherichia coli*	*Shigella boydii*
GCF_012221565	*Escherichia coli*	*Shigella boydii*
GCF_013374815	*Shigella sonnei*	*Escherichia coli* 02
GCF_013394495	*Shigella boydii*	*Shigella flexneri*
GCF_016026215	*Escherichia coli*	*Shigella boydii*
GCF_016026235	*Escherichia coli*	*Shigella boydii*
GCF_016026375	*Escherichia coli*	*Shigella boydii*
GCF_019793575	*Shigella flexneri*	*Escherichia coli* 02
GCF_022353545	*Shigella flexneri*	*Escherichia coli* 02
GCF_022353565	*Shigella flexneri*	*Escherichia coli* 02
GCF_022353605	*Shigella flexneri*	*Escherichia coli* 02
GCF_022353685	*Shigella flexneri*	*Escherichia coli* 01
GCF_022353745	*Shigella flexneri*	*Escherichia coli* 01
GCF_022353785	*Shigella flexneri*	*Escherichia coli* 01
GCF_022353885	*Shigella flexneri*	*Escherichia coli* 02
GCF_022353945	*Shigella flexneri*	*Escherichia coli* 01
GCF_022353965	*Shigella flexneri*	*Escherichia coli* 01
GCF_022354005	*Shigella flexneri*	*Escherichia coli* 01
GCF_022354025	*Shigella flexneri*	*Escherichia coli* 01
GCF_022354065	*Shigella dysenteriae*	*Escherichia coli* 09
GCF_022354085	*Shigella dysenteriae*	*Escherichia coli* 09
GCF_022354185	*Shigella flexneri*	*Escherichia coli* 01
GCF_022354205	*Shigella flexneri*	*Escherichia coli* 01
GCF_022354225	*Shigella flexneri*	*Escherichia coli* 01
GCF_022354245	*Shigella flexneri*	*Escherichia coli* 02
GCF_022354305	*Shigella flexneri*	*Escherichia coli* 01
GCF_022354325	*Shigella flexneri*	*Escherichia coli* 02
GCF_022354345	*Shigella flexneri*	*Escherichia coli* 02
GCF_022354585	*Shigella flexneri*	*Escherichia coli* 01
GCF_022354645	*Shigella flexneri*	*Escherichia coli* 02
GCF_022354685	*Shigella flexneri*	*Escherichia coli* 02
GCF_022354705	*Shigella flexneri*	*Escherichia coli* 02
GCF_022493955	*Shigella flexneri*	*Escherichia coli* 01
GCF_022493995	*Shigella flexneri*	*Escherichia coli* 01
GCF_022494015	*Shigella flexneri*	*Escherichia coli* 01
GCF_022494035	*Shigella flexneri*	*Escherichia coli* 01
GCF_022494095	*Shigella flexneri*	*Escherichia coli* 01
GCF_022494115	*Shigella flexneri*	*Escherichia coli* 01
GCF_022494135	*Shigella flexneri*	*Escherichia coli* 01
GCF_022494155	*Shigella flexneri*	*Escherichia coli* 01
GCF_022494175	*Shigella flexneri*	*Escherichia coli* 01
GCF_022494355	*Shigella flexneri*	*Escherichia coli* 01
GCF_022494415	*Shigella flexneri*	*Escherichia coli* 01
GCF_022494435	*Shigella flexneri*	*Escherichia coli* 02
GCF_022494455	*Shigella flexneri*	*Escherichia coli* 01

Each proposed phylogroup was named after the most frequent species name in the group.

A prior report, founded on mash distances and supported by further analyses, suggested that the *E. coli* + *Shigella* complex, could be divided into 14 phylogroups [26], fewer than our suggested 25 phylogroups above. Therefore, for comparison purposes, we found a mash distance threshold that would cut the hierarchical cluster into 14 groups (0.023). Only two of these were *Shigella* groups, in apparent agreement with the two *Shigella* phylogroups suggested before [26], though our two *Shigella* groups were separated in the hierarchy by clean *E. coli* groups (Fig 3, mid circle; S3 Table), rather than next to each other as in the prior study [26]. One of the *Shigella* groups was mainly composed of genomes named after all four species: *S. flexneri*, *S. sonnei*, *S. boydii* and *S. dysenteriae*, in order of abundance (Fig 3, mid and inner circles; S3 Table). The second *Shigella* cluster was composed exclusively of ten *S. dysenteriae* genomes. Three of the remaining twelve *E. coli* groups contained genomes named after *Shigella* species (Fig 3, mid circle; S3 Table).

The results presented in this section suggest that, though most genomes showed phylogenomic coherence matching their species labels, 48 genomes in the complex appear to be mislabelled (Table 2). Though our 25 apparent phylogroups seemed coherent, deciding whether the *Shigella* clusters should correspond to two or more phylogroups might require further analyses that are beyond the scope of this research.

## Conclusion

As mentioned, MinHash algorithms are already widely used. Our results should increase the confidence in such uses. For example, at the Bacterial and Viral Bioinformatics Resource Center (BV-BRC), it is used to rapidly identify similar genomes [27], and now scientists using this resource can be more confident that close relatives to their bacteria of interest will be properly identified. MinHash, mostly via mash, has also being part of the organization of huge genome data collections [28, 29]. In these works, the programs have been used for a first pass gathering of similar genomes, to later use ANI, or other more comprehensive methods, to finalize their organization into species and strains. MinHash methods have also been used in attempts at overall prokaryotic taxonomy [30], again, as a first attempt to gathering similar genomes to later analyse with more comprehensive methods. In all these cases, it is possible to infer that MinHash alone might have been enough to do the job, or, at least, that its initial role missed nothing that a more comprehensive, albeit slow, method would have accomplished. Our results should make the use of MinHash algorithms for the confident identification of bacteria in all kinds of settings, from epidemiological to industrial.

Overall, our results suggest that using fast sketch-based estimates of genome similarity, can be as accurate for bacterial delimitation as whole-genome Average Nucleotide Identity. Therefore, these estimates should suffice for further determination of the role that genome similarity should play in bacterial taxonomy and identification.

## Supporting information

S1 Table Enterobacterales genomes annotated to the species level. https://www.doi.org/10.6084/m9.figshare.22680310.
(TSV)

S2 Table Distances between Enterobacterales genomes by Average Nucleotide identity and MinHash (mash, dashing). https://www.doi.org/10.6084/m9.figshare.22680322.
(TSV)

S3 Table Clusters in the *Escherichia coli* + *Shigella* complex. https://www.doi.org/10.6084/m9.figshare.22680313.
(TSV)
